# Maintaining Digestive Health in Diabetes: The Role of the Gut Microbiome and the Challenge of Functional Foods

**DOI:** 10.3390/microorganisms9030516

**Published:** 2021-03-03

**Authors:** Eugenia Bezirtzoglou, Elisavet Stavropoulou, Konstantina Kantartzi, Christina Tsigalou, Chrysa Voidarou, Gregoria Mitropoulou, Ioanna Prapa, Valentini Santarmaki, Vasiliki Kompoura, Amalia E. Yanni, Maria Antoniadou, Theodoros Varzakas, Yiannis Kourkoutas

**Affiliations:** 1 Laboratory of Hygiene and Environmental Protection, Department of Medicine, Democritus University of Thrace, 68100 Dragana, Alexandroupolis, Greece; empezirt@yahoo.gr; 2 Centre Hospitalier Universitaire Vaudois (CHUV), rue du Bugnon, 1011 Lausanne, Switzerland; 3 Department of Infectious Diseases, Central Institute, Valais Hospital, 1950 Sion, Switzerland; 4 Department of Medicine, Nephrology Clinic, Democritus University of Thrace, 68100 Dragana, Alexandroupolis, Greece; kkantart@med.duth.gr; 5 Laboratory of Microbiology, Department of Medicine, Democritus University of Thrace, 68100 Dragana, Alexandroupolis, Greece; ctsigalo@med.duth.gr; 6 Department of Public Health P.U., Prefecture of Epirus, 47132 Arta, Greece; xvoidarou@uoi.gr; 7 Laboratory of Applied Microbiology and Biotechnology, Department of Molecular Biology and Genetics, Democritus University of Thrace, 68100 Dragana, Alexandroupolis, Greece; grigoriamitropoulou@gmail.com (G.M.); ioannaprap@gmail.com (I.P.); valentina.2@windowslive.com (V.S.); vickykom20.70@gmail.com (V.K.); ikourkou@mbg.duth.gr (Y.K.); 8 Laboratory of Chemistry-Biochemistry-Physical Chemistry of Foods, Department of Nutrition and Dietetics, Harokopion University, 17671 Athens, Greece; ayanni@hua.gr; 9 Dental School, National and Kapodistrian University of Athens, 11527 Athens, Greece; mantonia@dent.uoa.gr; 10 Department of Food Science and Technology, University of the Peloponnese, 24100 Kalamata, Greece

**Keywords:** diabetes, fermented foods, functional foods, gut microbiome, digestive health, nutrition, probiotics, prebiotics

## Abstract

Over the last decades, the incidence of diabetes has increased in developed countries and beyond the genetic impact, environmental factors, which can trigger the activation of the gut immune system, seem to affect the induction of the disease process. Since the composition of the gut microbiome might disturb the normal interaction with the immune system and contribute to altered immune responses, the restoration of normal microbiota composition constitutes a new target for the prevention and treatment of diabetes. Thus, the interaction of gut microbiome and diabetes, focusing on mechanisms connecting gut microbiota with the occurrence of the disorder, is discussed in the present review. Finally, the challenge of functional food diet on maintaining intestinal health and microbial flora diversity and functionality, as a potential tool for the onset inhibition and management of the disease, is highlighted by reporting key animal studies and clinical trials. Early onset of the disease in the oral cavity is an important factor for the incorporation of a functional food diet in daily routine.

## 1. Introduction-Diabetes as a Disease

The purpose of this non-systematic review is to discuss diabetes and see how the gut microbiome interacts with diabetes, describing the main mechanisms. The incorporation of fermented foods in order to maintain digestive health is very critical and we will demonstrate how this is achieved by description of animal and health models. This tool will aid in the prevention and management of this disease that so many people suffer nowadays.

According to the World Health Organization (WHO) and the International Diabetes Federation (IDF), the prevalence of diabetes has risen continuously over time from 108 million in 1980 [1] to approximately 463 million adults (20–79 years) living with diabetes in 2019; by 2045 this will rise to 700 million [2]. Furthermore, 4.2 million deaths were caused by diabetes and it costed at least 760 billion dollars (USD) in health expenditure in 2019. The global disease prevalence in adult population has risen from 4.7% to 8.5% during the years from 1980 to 2014 [1].

T2DM is described as a heterogeneous group of disorders. Most diabetic patients suffer from type 2 diabetes mellitus (T2DM) as a result of excess body weight and sedentary lifestyle. It is characterized by decline in insulin-producing *β*-cells, progressive peripheral insulin resistance and increased hepatic glucose production [3,4]. Without any doubt, diabetes etiology is strictly related to environmental and hereditary factors [5,6]. Women developing gestational diabetes mellitus (GDM) following pregnancy have high risk of developing Τ2DM [7,8]. In pregnant women, GDM is closely associated with phenotypes of metabolic disorders and more specifically obesity, insulin resistance and low-grade inflammation [7]. Chronic and low-grade inflammation is the hallmark of metabolic diseases, along with lipotoxicity-mediated production of cytokines, recruitment and phenotype changes of B and T cells, which promote macrophages infiltration into adipose tissue [9,10].

In T1DM, there is destruction of *β*-cells and little or no insulin is produced [11,12]. Viruses also seem to induce type 1 diabetes mellitus (T1DM) via molecular mimicry mechanism [13,14,15].

Symptoms may be almost identical in all types of disease including high blood glucose rates, polydipsia, polyuria, neuropathy, kidney failure, blindness, stroke, heart attack and limb amputation [16]. Oral manifestations are among the first to be seen in the human body and need to be early related to DM. Periodontal disease, periapical lesions, xerostomia and taste disturbance were more prevalent among diabetic patients as authors stated in a recent meta-analysis [17].

Heredity, ethnicity and feeding habits seem to increase diabetes burden. Asiatic populations for example, showed a lower prevalence of disease when compared with European populations [16]. Socio-economic status is another factor related to the disease prevalence. The increased rate of T2DM in urban Western societies has been linked to food selection, obesity, physical inactivity and lifestyle [18]. In this vein, urbanism in India favors the increasing rates of diabetes from 2.1% in 1970 at 11.6% in 1996 in adults [19]. Accordingly, a recent cross-sectional study showed variations in the diabetes picture between India’s different states [20] ranging from 4.3% to 11.8% [20]. Low socio-economic status groups living in disadvantaged urban areas showed a higher prevalence of diabetes [20]. Surprisingly, Germany had the highest prevalence rates in Europe in 2019 (15.3%) followed by Portugal (14.2%) and Malta (2.2%), while at the other end Ireland showed 4.4% [21]. However, most European countries showed a rate ranging from 6.3% to 10% [21]. Agglomeration index was positively associated to the diabetes prevalence while urban percentage was negatively associated. It seems that the model of urban development and not the urbanization as such determine disease prevalence [21]. 

**Figure 1:**
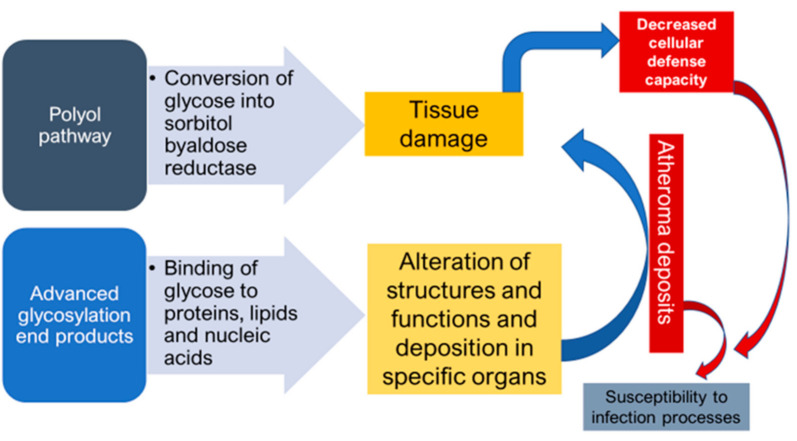
Mechanisms of formation of diabetic complications.

DM as a ‘’flame within’’ causes different complications mainly through two mechanisms. Firstly, the polyol pathway converts glucose into sorbitol, by aldose reductase enzyme, that causes tissue damage and numerous diabetic complications. Secondly, the formation of advanced glycosylation end products (AGEs), due to binding of glucose to proteins, lipids and nucleic acids, results in the alteration of structures and functions, in addition to its deposition in specific organs that causes various complications. Atheroma deposits are formed in cells, which accumulate in the basal membrane and lumen causing decreased cellular defense capacity and impaired polymorphonuclear leukocyte response. This makes diabetic patients more susceptible to infections especially by anaerobic bacteria due to the reduction of oxygen diffusion through the capillary wall [17] (Figure 1).

## 2. Microbiome and Diabetes

### 2.1. Microbiome at a Glance

The human newborn starts life with a likely colonized gut [22,23,24]. Bacteria coming from the environment, hospital staff in case of caesarian section [25], or from the maternal vaginal flora [26] colonize the newborn. However, personal habits, infections, stress, hormonal status, antibiotics and vaccination seem to be crucial factors for the establishment of the bacterial microflora [27], which is coined the term “microbiome” as genomes of the microbes are also involved. This term was firstly proposed by the Nobel Laureate Joshua Lederberg [27]. The microbiome includes bacteria-bacteriome, viruses-virome and fungus-mycobiome with various interactions among these -biomes and the host [15].

Microbial communities are characterized by complex microbial inter-dialogue and network patterns of unique microbiomes. This fact gained attention by many scientists, who proceeded to the characterization of the microbial communities profile in health and disease by the aid of new technologies, and specifically the 16S rRNA sequencing, in order to identify their complexity [28]. Metagenomics Whole Genome Shotgun (WGS) sequencing substantially conferred towards this aim. The *Human Microbiome Project* (USA) [28], as well as *the metaHIT Consortium* (Europe) [29] were dedicated to the characterization of major body sites microbiota in health as a hallmark stamp to compare with shifts occurring in disease states [30]. As a result, the intestinal flora is currently considered the largest and most complex organ composed of more than 1000 bacterial species and moreover many studies have pointed out the relation of various metabolic and immune disorders with intestinal microbial dysbiosis [31]. 

The importance of diet in shaping the human gut microbiome is stated since the very early age. The role of the healthy microbiota is crucial on the different metabolic processes, such as the breakdown of dietary fibers to short-chain fatty acids, breakdown of mucins, biosynthesis of amino acids and vitamins, and production of neurotransmitters and hormones [32]. The effect of feeding modes in the shape of gut microbiota is extensively discussed in multiple studies since the newborn birth to the adult age [33,34,35,36]. Important differences in microbial populations seem to be associated with dietary habits. There is also information derived from observational studies between globally distinct populations, such as children in rural Africa (Burkina Faso; BF) versus urban Europe children [37]. BF children developed a microbiota rich of bacteria belonging to the phylum *Bacteroidetes* and depletion of *Firmicutes*, while European recipients hosted more *Firmicutes and Proteobacteria.*

Gut microbiota own an essential role in maintaining host physiology, as they are involved in the digestion of several nutrients, development of the immune system and stimulation of immune responses against pathogens [9,38]. The ‘hygiene hypothesis’ implicating smaller families and lower exposure to child infections as an explanation for atopic disorders has been debated over numerous studies [39,40] In this vein, microbiota co-evolves with the human immune system and promotes a normal immune development [41]. A body of evidence for the key role of intestinal microbiota came into view after comparing host genes expression involved in immune responses, barrier function and energy homeostasis in germ-free and conventionally raised mice [42]. In most cases, enhancement of conditional pathogenic bacteria population, like *Bacteroides, Enterococcus, Ruminococcus* and *Desulfovibrio* over beneficial flora has been linked with lower levels of short chain fatty acids (SCFAs) and bile acids, as well as disruption of normal intestinal barrier function and endotoxemia. Thus, the mechanisms underlying the crucial role of commensal bacteria in human homeostasis maintenance are highlighted [43].

### 2.2. Mechanisms Connecting Gut Microbiota with Occurrence of T2DM 

#### 2.2.1. Short-Chain Fatty Acid Contribution

Short-chain fatty acids (SCFAs) are abundant in the intestine (mainly in the colon), and are produced by bacteria of the genus *Bacteroides, Clostridium, Bifidobacterium, Eubacterium* and *Streptococcus,* during fermentation of polysaccharides. Apart from maintaining low pH in the lumen and inhibit the growth of harmful bacteria, SCFAs act as an energy source for intestinal epithelial cells [38]. Additionally, they have a major role in the physiological intestinal anti-inflammatory response and the activation of multiple pathways signaling for fat and glucose metabolism, through *G*-protein coupled SCFAs receptors [9]. Especially, SCFAs inhibit histone deacetylase action and repress the activity of nuclear transcription factor NF-κB, thus affecting the production and release of pro-inflammatory molecules from neutrophils and macrophages [44]. Decreased levels of SCFAs, because of intestinal microbial imbalance, are associated with extended release of IL-2, IL-8 and TNF-a, which promote intestinal inflammation. Additionally, the anti-inflammatory cytokine IL-10 is capable of inhibiting synthesis of pro-inflammatory cytokines, such as IFN-γ, IL-2, IL-3, and TNFα released by macrophages and Th1 cells, while low levels of IL-10 have been reported in patients with T2DM and metabolic syndrome [45]. Similarly, fewer regulatory T cells, which produce IL-10, have been associated with chronic inflammation and it is well known that SCFAs promote T-cell differentiation through inhibition of histone deacetylase in T-cells and regulation of phosphorylation of the ribosomal protein S6 [44,46]. The junction between decreased levels of SCFAs and occurrence of T2DM is confirmed by abnormalities in lipid and glucose metabolism, which are regulated by GPR41 and GPR43 receptors. Acetic, propionic and butyric acids are ligands of the receptors and binding on the GPR41 receptor triggers the regulation of pancreatic *β*-cells and insulin secretion. Moreover, the peptide tyrosine-tyrosine (PYY) is normally released from differentiated gastrointestinal epithelial L-cells, in response to feeding and regulates appetite [47]. Partial activation of GPR41 receptors, due to low production of SCFAs, results in insufficient secretion of PYY and increased food intake. In combination with decreased insulin secretion by islet *β*-cells, the pathological condition of insulin resistance is established [9,47]. Furthermore, reduced appetite can be mediated by butyrate and propionate via induction of leptin expression from adipocytes [47]. Among SCFAs, butyric acid is considered as an important molecule for triggering pancreatic secretion. Reduced counts of butyric acid-producing bacteria result in decrease of glucagon and insulin levels, but increased blood glucose levels. In line with GPR41 receptors, SCFAs are ligands to GPR43 receptors, which stimulate signaling pathways regulating energy intake and fat metabolism [9].

#### 2.2.2. Lipid Metabolism

Intestinal microbial dysbiosis has also a negative impact on bile acids circuit and down activation of bile acid receptors is claimed to induce insulin sensitivity, increased appetite and body weight [48,49]. Specifically, liver cells produce primary bile acids via cytochrome P450-mediated oxidation of cholesterol and once secreted into the lumen, intestinal bacteria metabolize them into secondary bile acids without glycine and taurine groups. Bile acids act as micelle-forming surfactants and have major contribution to lipid and fat-soluble vitamins absorption and digestion [47]. Regarding unconjugated bile acids as signal molecules, their action resembles that of hormones and activate many nuclear receptors, particularly farnesol X-receptor (FXR) and G-protein-coupled bile acid receptor 5 (TGR5). In general, active FXR receptors suppress bile acids synthesis from cholesterol and lipogenesis, thus regulating hepatic triglyceride levels. Low levels of bile acids cut down signaling through FXR receptors and high levels of blood cholesterol and glucose have been reported [50,51,52]. Many TGR5 receptors are expressed in small intestine, liver or stomach, also in monocytes and macrophages cells, as they have an important role in energy homeostasis and glucose metabolism, and may possess an anti-inflammatory effect [10,53]. Activation of TGR5 receptors on L-cells promotes secretion of glucagon-like peptide-1 (GLP-1), a key insulin sensitizing and trophic hormone. In pancreatic β-cells, insulin secretion pathway initiates when active TGR5 receptors induce cAMP production and high levels of cAMP subsequently stimulate Epac (Exchange protein directly activated by cAMP), which in turn results in phosphoinositide hydrolysis and insulin release [54]. In a recent study [49], it was pointed out that TGR5 can induce GLP-1 secretion from pancreatic α-cells via an Epac-mediated protein kinase A-independent signaling pathway. GLP-1 is thought to be an important regulating hormone of glucose homeostasis, as it triggers insulin release, inhibits glucagon release and as a result low blood glucose has been reported [49,53]. Interestingly, Perino et al. [53] suggested that TGR5 signaling is involved in adipose tissue protection from inflammation, through inhibition of macrophages migration and decreased lipopolysaccharides-induced chemokine expression. Using Tgr5-knockout obese mice, they also revealed that chemokine reduction was mediated by AKT-dependent activation of mTOR complex 1 axis and differential translation of the liver inhibitory protein (LIP) in macrophages [53]. Many studies have also investigated the implications of TGR5 in obesity [48,55]. Bile acids mediated activation of TGR5 receptors promotes energy expenditure in brown adipose tissue and has a major contribution in balancing energy intake and lipid metabolism. The potential role of TGR5 in modulating inflammation was confirmed in TGR5 knockout mice. Bensalem et al. [48] investigated the inflammatory status in TGR5-/- obese mice. Noticeably, they observed higher circulating levels of lipopolysaccharides and increased IL-6, IL-1β, as well as TNFα mRNA expression in intestine, white adipose tissues and liver of TGR5-/- obese mice compare to WT obese mice. Obviously, intestinal dysbacteriosis and reduced levels of bile acids attenuate signal cascades transduction via FXR and TGR5 receptors and have an important role in occurrence and progression of metabolism diseases.

#### 2.2.3. Disruption of Intestinal Barrier Function and Endotoxemia

As previously mentioned, intestinal microbiota plays a pivotal role in the development and maintenance of the mucosal immune system, but on the other hand, can trigger chronic and low-grade inflammatory responses at metabolic disorders. Specifically, bacterial lipopolysaccharides (LPS) are the major components of the outer membrane of Gram-negative bacteria, such as Proteobacteria, normally secreted during membrane vesicle trafficking activity, although they serve as an endotoxin. Increased level of Proteobacteria is a well-established intestinal microbial shift in obesity and T2DM caused by high fat diet [38,56]. Dissociated endotoxins can be found in the bloodstream, a situation defined as metabolic endotoxemia, a contributory factor to the occurrence and progression of metabolic diseases [57]. Released LPS molecules can infiltrate adipose tissues, initiating many signal cascades, which involve innate immune responses, dysregulation of glucose and lipid metabolism [9,57,58]. Briefly, LPS bind to CD14 receptor expressing macrophages and via Toll-like receptor 4 (TLR4) signaling, a local secretion of pro-inflammatory key cytokines including TNF-α, IL-12, IL-6, IL-1β, INFβ and INFγ occur [58,59]. Active TLR4 stimulate the mitogen-activated protein kinase (MAPK) pathway and in particular, extracellular signal-regulated protein kinases 1 and 2 (ERK1/2), c-Jun-N-terminal kinases (JNK) and p38 signals transduction, which are involved in the activation of insulin signaling pathways [59]. Shi and colleagues [60] investigated the connection of metabolic endotoxemia and obesity-associated insulin resistance. Mice consuming a high-fat diet and lacking *TLR4* were protected from insulin resistance and lipid infusion did not activate NF-κΒ signaling in adipose tissue [60]. 

It is worth mentioning that increased systemic levels of LPS, or other metabolic products, are directly related with disruption of gut barrier function and permeability. LPS can cross intestinal mucosa via infiltrating chylomicrons which are lipoproteins responsible for the absorption of dietary triglycerides and cholesterol [61]. Intestinal barrier function refers to the ability to absorb dietary nutrients, while restricting undesirable luminal contents within the gut and is a heterogeneous entity composed by physical, chemical, microbial and immune elements [9]. Concerning microbial barriers, dietary habits play an important role in the modulation of intestinal microflora and excess consumption of fat causes destruction of microbial barrier, which in turn favors the microbial translocation and colonization of intestinal mucosa by pathogenic bacteria [61]. Commensal microflora influence barrier function by stimulating epithelial cell proliferation and by producing SCFAs, which serve as energy source [62]. The epithelial layer (physical barrier) of intestinal barrier is bound by tight junctions composed of many proteins, named occludins, claudins, junctional adhesion molecule and zozula occludens. Lower levels of junction proteins occluding and tricellulin and higher levels of LPS and zonulin were observed in obese people compare to lean controls [63]. 

#### 2.2.4. Inflammation and the Immune System

As previously discussed, diabetic recipients developed a constant systematic inflammation with high levels of pro-inflammatory cytokines [TNF-a, IL-6, b kinase inhibitor (IKKb) and Jun N-terminal kinase (JNK)], having negative impact on insulin [64]. Lactic acid bacteria have antioxidant capacity and can target the inflammatory status in diabetes mellitus, improve prevention and alleviate diabetes disease symptoms in animal models [65,66]. However, this effect is strain-dependent. *L. rhamnosus* strains showed enhanced effect compared to *Bifidobacterium* strains in the regulation of the glycolipid metabolism, as well as on gut microbiota improvement [66]. It is worth noting that lactic acid strains which presented hypoglycemic effects displayed a positive role in reducing insulin resistance by producing SCFAs and thus alleviation of inflammation status [66].

As analyzed above, the gut microbiota plays an important role in the balance of our health status with its metabolic profile. T2DM is linked to important alterations of the gut microbiota, due to the occurring dysbiosis. Particularly, T2DM is linked to a chronic inflammation status in fat tissue and maladjusted metabolism [67]. Yet, obesity enhances the problem, as it is associated to a pro-inflammatory cytokine production due to the inflammation. Immunological obesity is linked to pro-inflammatory cytokine secretion, immune cell infiltration and disrupted function of tissues involved in glucose homeostasis [67]. Specifically, lipid metabolism disorder parallel obesity and can impede insulin signaling. 

In addition, pattern recognition receptors (PRRs) activate the inflammation status and the presence of nutritional free fatty acids (FFAs), which display a negative impact on insulin target tissues in obesity [60,68]. Those circulating FFAs are increased in obesity and induce TLR4 signaling in macrophages and adipocytes and tissues inflammation [60,68]. Yet, it is believed that accumulation of lipids such as, diacylglycerol (DAG) and ceramides impede insulin action [69].

The expression of pattern recognition receptors (PRRs) is stimulated in the human cells during inflammation processes [70]. The most known PRRs are TLRs, which are membrane glycoproteins. TLR4 signaling in cells is critical for the inflammation process and probiotics reduce inflammation by limiting the expression of TLR4 [71] and thus modulating beneficially the microbiota. TLRs are found on cellular surfaces in increased amounts in individuals with diabetes, obesity and metabolic syndrome [72].

The gut microbiome seems to regulate TLR-mediated insulin resistance as experimental studies in mice deficient-TLR5 developed metabolic syndrome and insulin resistance due to dysregulation of IL-1β signaling [73]. More studies on animal models TLR2-deficient showed similar results as mice developed obesity, insulin resistance and glucose intolerance. The gut microbiome of these mice showed abundance of *Firmicutes* and reduced levels of *Actinobacteria* of the genus *Bifidobacterium* [74]. Moreover, low amounts of the beneficial *Bifidobacterium* confer on increasing gut permeability and thus can lead a leaky gut status with high endotoxins levels as LPS. In this vein, the immune system recognizes LPS and triggers TLR signaling and inflammation followed by insulin resistance and glucose intolerance. Accordingly, healthy mice assigned fecal microbiota transfer from animals having metabolic syndrome, they underwent the same progression of diseases states reversible by antibiotics administration [73].

As stated, the gut microbiome regulates TLR, hence the loss of TLR2 in mice shifts considerably the microbial flora profile. In contrast, enhanced expression of TLR2 is observed in patients with metabolic syndrome and diabetes [75]. 

#### 2.2.5. Microbiota Traits in DM

The key role of the gut microbiota is underpinned in multiple studies of T1DM and includes an increased ratio of duodenal *Bacteroidetes*/*Firmicutes* [76], an overgrowth of opportunistic pathogens [77] and decreased microbial diversity [78].

By the use of novel technologies, such as the deep tag-encoded sequencing, increased amounts of *Firmicutes* and *Clostridia* were reported in diabetic patients [79]. Moreover, the ratios of *Bacteroidetes* to *Firmicutes* and *Bacteroides–Prevotella* to *C. coccoides–Eubacterium rectale* groups were found to parallel FBG levels [79]. Interestingly, the *Betaproteobacteria* amounts were increased in diabetic individuals [79].

As mentioned previously, TLR signaling in cells is crucial for the inflammation process and the maintenance of tissue integrity [80]. TLR signaling is expressed through the adapter protein MyD88 and the lack of MyD88 prevents from vascular complications and atherosclerosis [80]. In this vein, mice models of T1DM fed with *L. johnsonii* and *L. reuteri* seems to impede diabetes development, due to the oxidative stress response and thus, lower pro-inflammatory cytokines amounts, such as interferon-γ are produced [81].

Arising out of the analysis of 30 billion nucleotide bases by the novel Illumina shotgun metagenome methodology, data investigation showed increased carbohydrate metabolism and stress responses [82]. Additionally, owing to the use of 16SrRNA shifts in the diversity of the microbiota, several microorganisms were revealed. Specifically, increased numbers of the phyla *Actinobacteria, Bacteroidetes a*nd *Proteobacteria* and the genus *Bacteroides* were found in diabetic, in contrast to the increased amounts of *Firmicutes, Fusobacteria, Tenericutes*, and *Verrucomicrobia* and low *Prevotella* levels were found in control individuals. Finally, lactate- and butyrate-producing bacteria were higher in healthy controls underlining their beneficial effect on the gut [82].

The human body disposes endogenous antioxidant mechanisms to keep the homeostasis. However, oxidation of the human cell occurs following exposure to physico-chemical and pathological conditions with the production of free radicals (reactive oxygen species, ROS) as a final result. Oxidative stress is installed when there is imbalance between ROS and antioxidant mechanisms. Disorders in lipid peroxidation, impaired glutathione metabolism and enzymatic function are taking place in diabetic patients. As people with T1DM showed increased levels of oxidative stress, development of major complications are occurring, such as cardiovascular disease, which remains the leading cause of morbidity and mortality in T1DM and T2DM [83]. It is also of note that the oxidative stress appears early in T1DM before autoimmunity development and *β-*cells damage [84]. Oxidative stress-related genes GPX1 and MPO in T2DM seem to be associated with the vascular complications by genetic predisposition [84]. Increased levels of ROS are involved in diabetic pathogenesis and oxidative stress through a dynamic correlation between nutrient excess and diabetes [85]. Evidence showed that obese individuals with insulin resistance have an elevated phylum *Firmicutes*/*Bacteroidetes* ratio compared to healthy people [86,87]. 

## 3. Functional Foods and Diabetes

### 3.1. The Challenge of Functional Foods 

Functional foods are foods that surpass classic nutrition and exert beneficial effects connected to their consumption [88] with optimization of markers related to the disease.

Undoubtedly, economic, political and social trends, together with technological advantages, lead people to migrate to towns [89]. As humans become more urban, the society meets the negative impact of urbanization due to the changing lifestyle. Living-conditions, restricted green space, scant sanitation, fat-food eating habits affect human health. Efforts have been made to prevent human disease development. Education and increased information on health issues make people shift their habits to more sustainable healthy solutions. In this vein, during the last decades, functional foods have gained particular attention, due to their relationship to nutrition and health [90].

Without any doubt, healthy nutrition preserves the intestinal ecosystem and beneficially affects metabolic regulation [91]. From another aspect, this interest in functional foods has played a significant role in the adoption of healthy habits, due to the increasing consumer health concerns [90,92]. 

Fermented foods were known since ancient times. Fermentation seems to be firstly used in the fertile crescent area of the Middle East in 6000 B.C. [92]. The term ‘acid milk’ was also mentioned in the Bible [90]. For centuries, the fermentation process was pragmatically used for food preservation and production in every culture. People foremost understood that fermentation enhance food shelf-life and improve organoleptic characteristics of foods [93]. As known, fermented foods contain edible microorganisms whose enzymes hydrolyze food polysaccharides, proteins and lipids to non-toxic products. As a result, a food transformation is taking place and ingredients beneficial for human health, such as SCFAs, are produced [94]. During the last years, higher throughput biotechnologies serve to promote the fermented food industrial production in a large scale and genome sequencing provided a global picture on the biodiversity of microorganisms in food fermentation processes [93]. New technologies tailor food with important characteristics through overexpression or disruption of respective metabolic genes [93]. Moreover, microbial interplay in the fermented food matrix affects food quality and safety, organoleptic properties and finally food digestibility and beneficial modulation of the host immune system [95].

A plethora of fermented products was developed, ranging from drinks to foods in every culture; kimchi in Asia, cassava in West Africa, kombucha tea in China, kefir yoghurt in Caucasian and Balkan countries, sauerkraut, pickles, apple vinegar in most Western countries. 

Nevertheless it is notable that most research focuses on the development of fermented dairy products. Metchnikoff was enrolled in a precursory research in the field of dairy fermentation in 1908. In his thesis under the title *“The Prolongation of Life”* evaluated the properties of lactic bacteria, specifically *Lactobacillus delbrueckii* subsp. *bulgaricus* and the longevity of Bulgarian farmers attributed to the consumption of fermented dairy products. Probiotics seem to prevent and reduce symptoms of multiple diseases, such as infections, autoimmune and allergic diseases and many others [96,97]. Even they are used as an adjunct therapy they maintain the balance of the intestinal microbiota [98].

Nevertheless, the country supporting vigorously the use of labeling in functional food products is Japan [99]. Since 1991, under the Foods for Specialized Health Use (FOSHU) label the Ministry of Health, Labor and Welfare in Japan issued a functional food regulation and launched their use in the market [99]. More than 200 functional products were branded under FOSHU legislation. In this vein, USA issued a regulation called “*Foods with Function Claims*” based on the Dietary Supplement Health and Education Act system (DSHEA) in 2015 [100]. This system seems to be more flexible in terms of health claims and in use of clinical protocols and thus launched functional foods market all over the world [100]. Registration of clinical studies must be under a University Hospital Medical Information Network (UMIN) protocol in both systems. While the FOSHU requires evidence that shows the reason for the minimum dosage from a dose-dependent study, as well as striking evidence based on the ways of action of the active compounds and the analytical methodology used, the American regulation system embeds on clinical studies proving a significant effect of functional foods when compared with a placebo intake group [100].

Functional food ingredients, such as probiotics, prebiotics, synbiotics and fermented as well, could amend the good condition of oral cavity and the activity of the gastrointestinal tract in a beneficial way. It is actually more than an axiom that their healthful effects are attributed to probiotic lactic acid bacteria (LAB) and *Bifidobacterium* [91].

The major effect of their intake is associated with the improvement of the host intestinal immune system through the ‘barrier effect’ and alleviation of the gut inflammatory response [97] through the production of immunoglobulin A (IgA) and balance between pro-inflammatory and anti-inflammatory cytokines [101]. It seems that diabetes is a disease related to the modern nutritional habits and lifestyle. Consumer’s interest in functional foods has increased due to their connection to health issues, such as the balance of gut microbiota and stimulation of the immune system [102]. 

As stated before, PRRs are of accrue importance for the deployment of the innate immune response [103]. Considering the above, therapeutic modulation of the gut microbiome via functional foods with probiotic properties may slow down the development of diabetes disease and its complications through a beneficial balance of the microbiota [104]. Knowledge gathered from animal models and human studies have shown that foods enriched with probiotics may impede postprandial hyperglycemia and adipose tissue and lipid metabolism occurring during diabetes inflammatory processes [104]. Furthermore, they seem to regulate dyslipidemia and insulin resistance status and reduce oxidative stress and inflammation [104]. In addition, they modify and regulate in a beneficial way the development of long-term diabetes complications, such as cardiovascular disease, neuropathy, nephropathy and retinopathy and oral manifestations [17,105] 

Many studies have examined the influence of specific eating patterns to the gut microbiome [106,107,108]. Animal studies stated that several *Lactobacillus* and *Bifidobacterium* species could prevent or even impede the severity of T2DM [109]. Additionally, studies in humans aimed to clarify metabolic shifts, oxidative stress and inflammation linked to diabetes [109].

Meta-analysis studies of multiple control trials stated that probiotics improved the fasting plasma glucose (FPG) and the glycosylated hemoglobin (HbA1c) in T2DM [110]. Similarly, probiotics given in people developing T2DM improve glycemic control [65].

Without any doubt, unraveling and exploring the involved microbial patterns and getting a better knowledge of the microbiota profile should clarify their role in health and disease and should lead to the development of more effective or even alternative therapeutic strategies and nutritional habits.

### 3.2. Animal Studies

A great amount of work has been dedicated in unveiling the health benefits of functional food ingredients, such as prebiotic fibers and probiotics on mechanisms regulating immunity system via modulation of the intestinal microbiota in diabetic animal models. There is an increasing number of studies concerning how functional foods can improve or be supplemented as an auxiliary treatment in metabolic disorders, such as obesity, atherosclerosis or diabetes. Functional foods seem to have additional physiological benefits and contribute to the reduction of the risk of chronic diseases beyond their basic nutritional functions [111,112]. 

The use of animal models in the study of diabetes, especially in the last decade, is a common practice [113]. The most widespread animals that are equipped in a variety of experimental protocols belong to the family of rodents, mainly mice and rats because they have a very similar genetic background to that of humans, as well as a short lifespan. Furthermore, they are highly productive, less expensive and easily treated by scientists and researchers than other animal models [114]. Literature findings are summarized in Table 1, concerning in vivo dietary supplementations in diabetic rodents and their outcomes on microbiome modulation and immunity regulation, all published in the last 10 years.

Xue et al. [115] employed T2DM rats and investigated the potential health effects of propolis. Interestingly, propolis led to lower fasting blood glucose (FBG), reduced insulin resistance and improved intestinal mucosal injury in ileum tissue. Microbiota of diabetic rats were normalized with predominant being the *Lactobacillus* genera that consists mainly of probiotic bacteria, whereas *Enterococcus*, *Clostridium*, *Turicibacter* and *Arthrobacter* were lower compared to the control group [115].

Another interesting study underlined the effects of pistachio nuts supplementation on amelioration of inflammation by lower inflammatory foci, IL-1β, CCL-2 gene expression and inflammatory markers (TNF-α and IL-1β) in Wistar rats under high fat diet (HFD). These obese pistachio-supplemented rats exhibited lower *Firmicutes*/*Bacteroidetes* ratio and increased health-related bacteria, such as *Parabacteroides*, *Dorea*, *Allobaculum*, *Turicibacter*, *Lactobacillus* and *Anaeroplasma*, while inflammation-associated genera like *Oscillospira, Desulfovibrio, Coprobacillus* and *Biophila* were decreased [116]. The health benefit of pistachios on the microbiome of T1DM rats has also been studied by our group [117]. In healthy animals receiving pistachios lactobacilli and bifidobacteria were found in increased numbers, as well as increased populations of the *Firmicutes* phylum were reported, but decreased amounts of *Bacteroidetes* phylum were recorded. 

Dietary supplementation on rats in HFD with barley or malt revealed decreased ratio of colonic *Firmicutes*/*Bacteroidetes* and increased numbers of *Actinobacteria* and *Verrucomicrobia* after barley supplementation. Furthermore, *Akkermansia*, *Ruminococcus*, *Blautia*, *Biophila*, *Turicibacter* and *Roseburia* genera were elevated after barley malt intake and shed some light in the manner of optimizing the health benefits of whole-brain barley products [118]. Cornstarch diet has been also associated with benefits in alleviating the adversity of diabetes mellitus. Studies that were performed in STZ-induced diabetic rats showed increased diversity of gut microbiota. It was observed a decrease of *Actinobacteria* and *Bacteriodetes* with increased abundance of *Firmicutes* [119,120]. In another similar study, the RA of these 6 OTUs, *Christensenellaceae* R-7 group, *Prevotella* 9, an unknown species of the *Prevotellaceae* family, *Prevotellaceae* UCG-001 and *Ruminococcaceae* UCG-005, and *Ruminococcus* 1, were all increased after starch feeding. Starch feeding also led to a reduction in RA of an uncultured species of the *Erysipelotrichaceae* family, *Escherichia*-*Shigella*, *Klebsiella*, an unknown species of the *Peptostreptococcaceae* family and *Turicibacter* [121].

Sane et al. [122] tested the effects of lone human milk administration in nod mice and underlined the prevention of diabetes onset and progression with elevated fecal *Bifidobacterium* and *Akkermansia* abundances and lower cecal *B. fragilis* and *E. coli*. Cecal and colonic *B. vulgatus* were enhanced by human milk intake [122].

Shikano et al. [123] used Green loofah *L. cylindrica* homogenate (LH) and fermented LH (FL) with *L. lactis* subsp. *lactis* Uruma-SU1 and *L. plantarum* Uruma-SU4, isolated from algal beach casts in a dietary supplementation experiment on a specific pathogen free (SPF) mice Kwl: ddY mice in high-fat diet. After FL consumption, TC, LDL-C and the ratio LDL-C/HDL-C were lower, whereas cecal *L. johnsonii* and *C. disporicum* were increased [123].

STZ-induced diabetic Wistar rats in high-fat diet (T2DM) were supplemented with fermented milk that was produced by inoculation of skim milk with probiotic cultures, *L*. *rhamnosus* NCDC 17 and *L. rhamnosus* GG, at 1% (*v/v*) [124]. Both probiotic treatments increased the population of total bacteria. *L. rhamnosus* NCDC 17 supplementation group had higher populations of *E. rectale*- *C. coccoides*, *Bacteroides*, lactobacilli and bifidobacteria. LGG and *L. rhamnosus* NCDC 17 decreased FGB and increased insulin levels and had a positive effect on glycosylated hemoglobin [124]. Free fatty acids levels and lipid profile were improved after HFD + *L. rhamnosus* NCDC 17 administration and triglycerides were reduced, whilst both probiotics increased HDL-C levels. Finally, *L*. *rhamnosus* NCDC 17 supplementation decreased expression levels of TNF-α and IL-6 genes and increased mRNA of the adiponectin gene [124].

A non-dairy fermented product with a combination of specific LABs and non-bitter beer yeast was administered to Zucker diabetic fatty (ZDF) rats, a model for T2DM associated with obesity [125]. Decreased glucose absorption was observed in treated group, along with decreased blood glucose. Microbial diversity was enriched after administration and *Firmicutes*, *Saturella*, *Proteus*, *Alistipes*, *Anaerococcus* were increased in the supplemented group, while *Streptococcacae*, *Anaerococcus* and *Streptococcus*, *Barnesiella* and *Blautia* were enriched in the control group. Hu et al. [126] showed that mixed fermentation by *L. fermentum* and *Saccharomyces cerevisiae* enhanced DNJ extraction efficiency from mulberry leaves. When implemented to STZ-induced diabetic mice, the extract seems to have relieved gut dysbiosis by promoting the growth of *Lactobacillus*, *Lachnospiraceae* NK4A136 group, *Oscillibacter*, *Alistipes* and *Bifidobacterium* [126]. At the same time, the growth of *Ruminococcaceae* UCG-014, *Weissella*, *Ruminococcus*, *Prevotellaceae* Ga6A1 group, *Anaerostipes*, *Klebsiella*, *Prevotellaceae* UCG-001 and *Bacteroidales* S24-7 group were significantly suppressed [126].

The efficacy of probiotics in diabetes relies on their ability to lower FBG and insulin levels in preclinical setting, as well as human trials [127]. VSL#3 is a probiotic product available in the market that contains strains of *Bifidobacteriaceae* (*B. longum, B. infantis* and *B. breve*), *Lactobacillaceae* (*L. acidophilus*, *L. paracasei*, *L. delbrueckii* subsp. *Bulgaricus* and *L. plantarum*) and *S. thermophilus.* When it was administrated to the non-obese diabetic (nod) mice model, where T1DM occurs as a result of insulitis, ameliorated diabetes progression took place, which was accompanied by reduced degree of insulitis in histological examination [128]. Inflammation markers were decreased as there was an inhibition of IL-1β expression and also enhancement of indoleamine-2, 3-dioxygenase (IDO) and IL-33 was documented [128]. An interesting outcome included the reduced differentiation of T-helper cells in autoimmune sites of the pancreatic lymph nodes (PLN), the site where the autoimmune response is regulated in T1DM [128]. The potential health benefits of probiotic administration were maximized with the increased *Lactobacillacae*, clostridia and *Rikenellaceae* after VSL#3 treatment and decreased abundance of *Bacteroidetes* strain S24-7 [128].

Other animal models, such as STZ-induced diabetic Wistar rats in HFD (T2DM), were utilized in exploring the supplementation with *L. plantarum* (probiotic), inulin (prebiotic) or in combination (symbiotic) [129]. Probiotic and synbiotic treatment led to an increase of *Firmicutes* phylum and of *Lactobacillales* family, while *Clostridiales, Enterococcaceae* and *Bacteroidales* were decreased [129]. Prebiotic treatment increased *Streptococcaceae* classification [129]. All treated groups were dominated with *Lactobacillus* genera and were characterized by enhanced *Lactobacillus/Firmicutes* ratio, whilst only the synbiotic supplementation increased specifically the probiotic *L. plantarum* population [129]. An improvement of oxidative stress status in hippocampus and prefrontal cortex, as well as a neuropsychological improvement and reversion of cognitive impairment were observed after symbiotic administration, underlying the variety of health benefits that probiotics can offer [129].

*L. rhamnosus* BSL and *L. rhamnosus* R23, when administered in STZ-induced diabetic Sprague-Dawley rats, led to elevated LAB levels after 30 days of probiotic supplementation and improved glucose tolerance and glucose control, as FBG was significantly reduced. After probiotic treatment, there was a decrease in TC and in atherogenic index [130].

Tian et al. [131], and Li et al. [132] used *L. paracasei* subsp. *paracasei* G15 and/or *L. casei* Q14 isolated from dairy food in STZ-induced diabetic Wistar rats in high-fat diet (T2DM) and concluded that glucose tolerance was restored and TC and triacylglycerol level was suppressed after 6 weeks of probiotic administration [131,132]. Hyperinsulinemia was ameliorated with insulin and glucagon levels being lower after probiotic ingestion and concentration of antidiabetic hormones GLP-1 and PYY was augmented after probiotic supplementation [131,132]. Furthermore, plasma LPS was reduced and a healthier intestinal microenvironment was achieved by improving intestinal barrier structure. The epithelial and mucosal structure was normalized and consisted of more integral mucosa [131,132]. Interleukins were also affected; specifically, IL-1β, IL-8 and IL-6 levels were diminished. The relative abundances of *Lactobacillus*, *Bifidobacterium*, *Clostridium leptum*, *Bacteroides* and *Prevotella* were increased after probiotic treatment [131,132]. 

Similar findings were observed in STZ-induced diabetic C57BL/6J mice in high-fat diet (T2DΜ) supplemented with *L. casei* CCFM419 [133]. Microbiota modulation was conducted with increased *Allobaculum* and *Bacteroides* genera and *Bacteroidetes* phylum abundances and decreased *Firmicutes* [133]. Post-probiotic ingestion, FBG and HBA1c and leptin levels were lower, 2-h postprandial blood glucose was reduced, and insulin sensitivity was improved by decreased fasting insulin concentration, as well as improvement of HOMA-IR value was noticed [133]. Inflammation was ameliorated with decreased levels of TNF-α, IL-6 and IL-10 [133]. Lipid control was conducted with reduced LDL-C and increased HDL-C levels [133]. Finally, there was a recovery of impaired islet cells and the expression of mRNAs of PI3K and GS, concerning the insulin resistance, was increased and GSK-3β mRNA expression was decreased [133].

*L. casei* Zhang, when administrated in STZ-induced diabetic Sprague-Dawley rats in high-fat sucrose diet (HFS) (T2DΜ), led to more abundant cecal *Bifidobacterium* and *Lactobacillus* genera and lower levels of *C. coccoides*–*E. rectale* group and *C. scindens* members [134]. Probiotic supplementation led to reduced endotoxin LPS production that had been induced by STZ and the onset and development of hyperglycemia in both fasting and postprandial 2 h blood glucose levels and OGTT levels was reversed [134]. What is more, pro-inflammatory cytokines (IFN-c and TNF-α) was inhibited after probiotic administration [134].

*L. rhamnosus* CCFM0528 provoked increased *Bacteroidetes* and decreased *Firmicutes* in phyla level and elevated *Bifidobacterium*, *Lactobacillus*, *Allobaculum* and *Bacteroides* genera in STZ-induced diabetic C57BL/6J mice in high-fat diet (T2DM) accompanied with amelioration of insulin resistance, glucose tolerance, FBG and postprandial 2-h blood glucose; TNF-α and IL-6 production were decreased and GLP-1 was increased [135].

All of the above-mentioned studies presented in detail in Table 1, provided similar results and reach to the same conclusion. Prebiotics, as well as probiotics and foods enriched with such ingredients can promote gut health via enhancement of the presence of beneficial bacteria genera and accumulation of advantages for the host immune system and metabolic regulation.

### 3.3. Human Studies

A range of food supplements are consumed by various groups of people with the aim of improving health. Several clinical studies have been conducted so far, especially with probiotics per se. There are also a small number of studies in which food products enriched with specific beneficial microbes are examined. These foods include mainly fermented milk products.

Allen et al. [136] evaluated the safety of a bacterial dietary supplement for the prevention of atopy in infants in a randomized, double-blinded, placebo-controlled trial. Two strains of lactobacilli (*L. salivarius* CUL61 and *L. paracasei* CUL08) and bifidobacteria (*B. animalis* subsp. lactis CUL34 and *B. bifidum* CUL20) with a total of 1 x 10^10^ colony-forming units were administered daily to women during the last month of pregnancy and to infants aged 0–6 months, with beneficial results [136].

Kassaian et al. [137] assessed the effects of probiotics and synbiotics on metabolic syndrome in individuals with prediabetes. One hundred and twenty adults with prediabetes were enrolled in a double-blind, placebo-controlled randomized parallel-group clinical trial [137]. Participants were randomized to a multi-species probiotic or inulin-based synbiotic or placebo. The potential benefits of using probiotic and synbiotic for metabolic syndrome management in prediabetes have been supported by the results, which provided an important strategy to combat metabolic syndrome-associated diseases [137].

Khalili et al. [138] divided forty patients with T2DM (*n*=20 for each group) into intervention (probiotic) and placebo groups. The intervention group received a daily capsule containing 10^8^ cfu of *L. casei* for eight weeks [138]. The patients in placebo group took capsules containing maltodextrin for the same time duration. In comparison with the placebo, *L. casei* supplementation significantly increased SIRT1 and decreased fetuin-A levels at the end of the trial in a way that improved glycemic response in subjects with T2DM [138]. Affecting the SIRT1 and fetuin-A levels introduced a new known mechanism of probiotic action in diabetes management [138].

Medina-Vera et al. [139] studied the effects of a functional food-based dietary intervention on fecal microbiota and biochemical parameters in patients with T2DΜ. In a placebo-controlled, randomized, double-blind study 81 patients with T2DM were divided into two 3-month treatment groups: one following a reduced-energy diet with a dietary portfolio (DP) comprising high-fiber, polyphenol-rich and vegetable-protein functional foods, the other taking a placebo (P) [139]. Patients with T2DΜ exhibited intestinal dysbiosis characterized by an increase in *Prevotella copri* [139]. Dietary intervention with functional foods significantly modified fecal microbiota compared with P group by increasing alpha diversity and modifying the abundance of specific bacteria, independently of antidiabetic drugs [139]. There was a decrease in *P. copri* and an increase in *Faecalibacterium prausnitzii* and *Akkermansia muciniphila*, two bacterial species known to have anti-inflammatory effects [139]. The DP group also exhibited significant reductions in areas under the curve for glucose, TC and LDL-C [139].

Sabico et al. [140] studied the effects of 6-months multi-strain probiotics supplementation in T2DM in a randomized, double-blind, placebo-controlled trial and concluded in the beneficial role of probiotics in inflammation, promising adjuvant anti-diabetes therapy [140]. 

Gut bacterial translocation to the blood may play an important role in the development of insulin resistance in T2DM. Sato et al. [141] investigated whether probiotics could reduce bacterial translocation and cause changes in the gut microbiota, in two groups of Japanese; the probiotic group that consumed *Lactobacillus casei* strain Shirota-fermented milk, and the control group administered no probiotics [141]**.** Probiotic administration reduced bacterial translocation and altered the gut microbiota in Japanese patients with T2DM [141].

In a randomized, double-blind, placebo-controlled study, participants were assigned into two groups: a probiotic group, consuming fermented milk containing *L. acidophilus* La-5 and *B. animalis* subsp *lactis* BB-12 (10^9^ colony-forming units/d, each) and a control group, consuming conventional fermented milk [142]. Probiotic consumption improved the glycemic control in T2DM subjects. However, the intake of fermented milk seems to be involved with other metabolic changes, such as decrease in inflammatory cytokines (TNF-a and resistin) and increase in acetic acid [142]. Furthermore, dietary intervention by consumption of yogurt with live probiotics and other dairy products seems to improve the antioxidant status and FPG levels in T2DM patients [143,144]. Table 2 shows representative human studies showing supplementation with fermented foods containing probiotics, prebiotics and synbiotics and the main beneficial outcomes in the human body (immune system and gut microbiome).

## 4. Conclusions

In conclusion, colonization of intestinal mucosa by beneficial microbes is related to the appropriate production of short chain fatty acids and bile acids, which have a positive effect in immune system development and can regulate signaling cascades involving energy intake and fat metabolism. On the other hand, microbial dysbiosis lead to chronic low-grade intestinal inflammation and insulin resistance, thus providing an advance for metabolic disorders. Evidence from animal and human studies stated probiotics as a modern approach with claimed health benefits. The inclusion of functional foods with probiotic properties in the daily dietary pattern consists of a positive approach with beneficial impact on human health and under this point of view could contribute to the improvement of the diabetes metabolic imbalance, preventive and therapeutic control of the disease. The involvement of oral and general health professionals in strategies aimed at identifying individuals at risk from diabetes through early manifestations and diet programs with functional foods should be maximized in order to retard the development of possible complications.

Our review deals with the role of intestinal microbiota in the pathogenesis of T2DM. As it is well known, gut microbiota keep a crucial place in the pathogenesis of T2DM by swaying body pro-inflammatory activity, insulin resistance and bile-acid metabolism. Undoubtedly, tempering the intestinal microbiota through the use of food ingredients showed improvement of the glucose metabolism and insulin resistance in the diabetic host. 

Notwithstanding, research into diabetes raises multiple inquiries to get better knowledge of the ‘dialogue’ between gut microbiota and diabetes T2DM. Animal models are valuable tools to explore these complex interactions and have responses on the pathophysiology of the disease and enable us with individualized therapies based on modulation of the intestinal microbiota in T2DM.

## Figures and Tables

**Table 1:** Animal models supplemented with food ingredients; the main beneficial outcomes in the immunity system and the after-effect alterations in intestinal microbiota.

Type of Compound	DietarySupplement	Animal Model	Outcomes In Immune System	Outcomes in Microbiota	Reference
**Prebiotics**	Propolis	STZ-induced diabetic Sprague-Dawley rats (T2DM), male	240 mg/kg propolis led to lower FBG levels. Insulin resistance reduced after propolis treatment. Propolis treatment could repair the intestinal mucosal injury	Overall structure of the gut microbiota in diabetic rats was shifted toward that in normal rats. *Lactobacillus* genera were predominant in the control and propolis treatment groups. Significant down regulation of the abundances of *Blautia*, *Fusicatenibacter* and *Clostridium*XlVa in the model group.	[115]
	High fat diet group supplemented with pistachio nuts	C57BL/6J (B6), male	Decreased amounts of TNF-α and IL-1βin serum (HFD-P vs HFD). Inflammatory foci, IL-1β, CCL-2 gene expression were lower in the liver of HFD-P vs HFD. Improvement of inflammation in obese mice.	Lower *Firmicutes*/*Bacteroidetes* in HFD-P *vs* HFD/ *Parabacteroides*, *Dorea*, *Allobaculum*, *Turicibacter*, *Lactobacillus* and *Anaeroplasma* increased with pistachio. *Oscillospira, Desulfovibrio, Coprobacillus* and *Biophila* reduced with pistachio supplementation.	[116]
	Pistachio nuts	STZ-induced diabetic Wistar rats (T1DM), male	-	Elevated levels of lactobacilli and bifidobacteria in jejunum, ileum and cecum of diabetic animals. Increased fecal lactobacilli and bifidobacteria counts and decreased enterococci after 4 weeks of pistachio diet (in healthy and diabetic animals).	[117]
	Whole grain barley / barley malt	Wistar rats, male	-	Lower *Firmicutes*/ *Bacteroidetes* increased *Actinobacteria* and *Verrucomicrobia*, after barley supplementation. *Akkermansia*, *Ruminococcus*, *Blautia*, *Biophila*, *Turicibacter*, *Roseburia* higher after barley malt intake.	[118]
	Corn starch diet with chlorogenic acid	Wistar rats, male	Decreased inflammation and fat deposition in the liver along with reduced plasma liver enzyme activities of obese rats.	Increased diversity of gut microbiota. Rats showed decreased abundance of *Actinobacteria* and *Bacteriodetes* with increased abundance of *Firmicutes*.	[119]
	Taro starch, beet juice, *L*. *plantarum* IS-10506	STZ-induced diabetic Sprague Dawley rats (T1DM), male	-	The RA of these six OTUs, *Christensenellaceae* R-7 group, *Prevotella* 9, an unknown species of the *Prevotellaceae* family, *Prevotellaceae* UCG-001 and *Ruminococcaceae* UCG-005 and *Ruminococcus* 1 were all increased after starch feeding.	[121]
**Probiotics**	*L. plantarum* (probiotic), inulin (prebiotic) or in combination (symbiotic)	STZ-induced diabetic Wistar rats (T2DM), male	Improvement of oxidative stress status in hippocampus and prefrontal cortex. Neuropsychological improvement.Cognitive impairment is reversed after symbiotic administration.	Probiotic and synbiotic treatment led to increase of *Firmicutes* phylum and in *Lactobacillales* family, while *Clostridiales* and *Bacteroidales* were decreased.	[127]
	VSL#3 containing *Bifidobacteriaceae*, *Lactobacillaceae*, and *Streptococcus thermophilus*, alone or in combination with Retinoic Acid.	Nod mice	Protection from diabetes progression Reduced degree of insulitis in histological examination. Inhibition of IL-1β expression. Enhancement of indoleamine-2,3- dioxygenase (IDO) and IL-33.	Increased *Lactobacillacae*, clostridia and *Rikenellaceae* after VSL#3 treatment. Decreased abundance of *Bacteroidetes* strain S24-7.	[128]
	*L. rhamnosus* BSL and *L*. *rhamnosus* R23	STZ-induced diabetic Sprague-Dawley rats (T2DM), male	Fasting blood glucose (FBG) was significantly reduced after probiotic administration. Improved glucose tolerance and glucose control after probiotic administration. Decrease in Total Cholesterol (TC)	Increased LAB levels after 30 days of probiotic supplementation	[130]
	*L. paracasei* subsp. *paracasei* G15 and/or *L. casei* Q14 isolated from dairy food alone or in combination with metformin	STZ-induced diabetic Wistar rats in high-fat diet (T2DM), male	Restored glucose intolerance in all treatment groups after 6 weeks. FBG decreased after 13 weeks in all treatment groups. Probiotics reduced plasma LPS. The lactobacillus and metformin treatments significantly reduced both IL-1β and IL-8 levels.	Separated clustering of microbiota in each group. Microbiota of group supplemented with *L. casei* Q14 was located near the healthy group.	[131]
	*L. paracasei* subsp. *paracasei* G15 and/or *L. casei* Q14 isolated from dairy food	STZ-induced diabetic Wistar rats in high-fat diet (T2DM), male	Restored glucose tolerance and suppressed total cholesterol and triacylglycerol (TAG) level, after 6 weeks of probiotic administration. Hyperinsulinemia was ameliorated with insulin and glucagon levels being lower after probiotic ingestion.	Abundances of *Lactobacillus* and *Bifidobacterium,Clostridiumleptum*, *Bacteroides*, *Prevotella*, were increased after probiotic treatment.	[132]
	*L.casei* CCFM419	STZ-induced diabetic C57BL/6J mice in high-fat diet (T2DM), male	Ingestion of *L. casei* CCFM419 led to lower FBG, reduced 2-h postprandial blood glucose. Improved insulin sensitivity by decreased fasting insulin concentration and HOMA-IR value. Decreased levels of TNF-α and IL-6 and IL-10. Reduced LDL-C and increased HDL-C levels.	The abundance of *Allobaculum* and *Bacteriodes* were increased after probiotic treatment. Decreased *Firmicutes* and increased *Bacteroidetes*.	[133]
	*L. casei*	STZ-induced diabetic Sprague-Dawley rats in high-fat sucrose diet (HFS) (T2DM), male	*L. casei* reduced the endotoxin LPS production induced by STZ. *L. casei* Zhang ingestion prevents from the onset and development of glycemia in both fasting and postprandial 2 h blood glucose levels and OGTT levels. Inhibition of pro inflammatory cytokines (IFN-c and TNF-α) after probiotic administration.	Caecal *Bifidobacterium* and *Lactobacillus* were more abundant in probiotic-treated rats in HFS diet than the plain HFS group. Higher *C. coccoides*–*E.rectale* group and C. scindens members in HFS than the probiotic HFS and control rats.	[134]
	*L. rhamnosus* CCFM0528	STZ-induced diabetic C57BL/6J mice in high-fat diet (T2DM), male	Amelioration of insulin resistance, glucose tolerance, FBG and postprandial 2-h blood glucose. Decreased TNF-α and IL-6. Increased GLP-1.	Increased *Bacteroidetes* and decreased *Firmicutes*.Increased *Bifidobacterium*, *Lactobacillus*, *Allobaculum* and *Bacteroides*.	[135]
**Fermented products**	Green loofah L. cylindrica homogenate (LH) and fermentedLH (FL) with *Lactococcus lactis* subsp. *lactis* Uruma-SU1 and *L. plantarum* Uruma-SU4, isolated from algal beach casts.	male Kwl: ddY mice in high-fat diet	TC, LDL-C, and the ratio LDL-C/HDL-C were lower after FL.	Caecal *L. johnsonii* and *C. disporicum* were increased through the consumption of fermented loofah.	[123]
	Fermented milk by inoculation of skim milkwith probiotic cultures (*L. rhamnosus* NCDC 17 and *L. rhamnosus* GG) at 1% (*v/v*)	STZ-induced diabetic Wistar rats in high-fat diet (T2DM), male	L. rhamnosus NCDC 17 supplementation decreased expression levels of TNF-α and IL-6 genes, increased mRNA of the adiponectin gene. LGG and L. rhamnosus NCDC 17 decreased FGB levels. Lower insulin levels after probiotic treatment.	Both probiotic treatments increased the population of total bacteria. HFD + *L. rhamnosus* NCDC 17 group had higher populations of *Eubacterium rectale*-*C. coccoides*, *Bacteroides*, lactobacilli and bifidobacteria.	[124]
	Non dairy fermented food product	Zucker diabetic fatty (ZDF) rats, male	Decreased glucose absorption in treated group. Decreased blood glucose with the FFP.	Enriched microbial diversity after FFP administration. Increased *Firmicutes*, *Saturella*, *Proteus*, *Alistipes*, *Anaerococcus* in FFP group and *Streptococcacae*, *Anaerococcus* and *Streptococcus*, *Barnesiella* and *Blautia* in control group.	[125]
	Mixed fermentation by *L. fermentum* and *Saccharomyces cerevisiae* was used to enhance DNJ extraction efficiency from mulberry leaves	STZ-induced diabetic Kunming mice, male	-	Relieved gut dysbiosis in diabetic mice by promoting the growth of *Lactobacillus*, *Lachnospiraceae* NK4A136 group, *Oscillibacter*, *Lachnospiraceae*, *Alistipes*, and *Bifidobacterium*.	[126]
**Other products**	Corn starch tea (instant or matcha)	Kunming mice, female		Particularly increased levels of *Coriobacteriaceae*, *Lactobacillaceae*, *Prevotellaceae* and *Bifidobacteriaceae*, and decreased *Bacteroidaceae*, *Ruminococcaceae*, *Helicobacteraceae* and *Enterobacteriaceae*.	[120]
	Human milk	Nod mice, female	Prevention of diabetes onset and progression.	Elevated fecal *Bifidobacterium* and *Akkermansia* by human milk. Cecal B. fragilis and *E. coli* lower after HM.Higher cecal and colonic *B. vulgatus* by human milk intake.	[122]

**Table 2:** Representative human studies showing supplementation with fermented foods containing probiotics, prebiotics and synbiotics and the main beneficial outcomes in the human body immune system.

Fermented Food/Probiotics	Outcome in the Immune System	Reference
fermented milk containing L. acidophilus La-5 and *B. animalis* subsp *lactis* BB-12	improved the glycemic control in T2DM subjects decrease in inflammatory cytokines (TNF-a and resistin) and increase of the acetic acid	[142]
The probiotic group consumed Lactobacillus casei strain Shirota-fermented milk	Probiotic administration reduced bacterial translocation and altered the gut microbiota in Japanese patients with T2DM	[141]
6-months multi-strain probiotics supplementation in T2DM	beneficial role of probiotics in inflammation, promising adjuvant anti-diabetes therapy	[140]
reduced-energy diet with a dietary portfolio (DP) comprising high-fiber, polyphenol-rich and vegetable-protein functional foods	Increase in *Faecalibacterium prausnitzii* and Akkermansia muciniphila, two bacterial species known to have anti-inflammatory effects. Dietary intervention with functional foods significantly modified fecal microbiota by increasing alpha diversity.	[139]

## Abbreviations

AGE	advanced glycosylation end products
BMS	burning mouth syndrome
D	type 2 iodothyronine deiodinase
DAG	diacylglycerol
DSHEA	Dietary Supplement Health and Education Act system
Epac	Exchange protein directly activated by cAMP
FBG	fasting blood glucose
FFAs	free fatty acids
FOSHU	Foods for Specialized Health Use
GDM	Gestational Diabetes Mellitus
GLP-1	glucagon-like peptide-1
TGR5	G-protein-coupled bile acid receptor 5
HFD	high fat diet
IKKb	b kinase inhibitor
IL	Interleukin
JNK	c-Jun-N-terminal kinases
LAB	lactic acid bacteria
LIP	liver inhibitory protein
LPL	lipoprotein lipase
LPS	lipopolysaccharides
MAPK	mitogen-activated protein kinase
PRRs	pattern recognition receptors
PYY	peptide tyrosine-tyrosine
SCFAs	Short-chain fatty acids
T1DM	type 1 diabetes mellitus
T2DM	type 2 diabetes mellitus
T3	active tri-iodothyronine
TLR4	Toll-like receptor 4
TMA	trimethylamine
TNF	Tumor necrosis facto
VLDL	very low-density lipoproteins
WGS	Whole Genome Shotgun
XR	Farnesol X-receptor
